# Influence of Culture Media on Microbial Fingerprints Using Raman Spectroscopy

**DOI:** 10.3390/s151129635

**Published:** 2015-11-24

**Authors:** Katarína Mlynáriková, Ota Samek, Silvie Bernatová, Filip Růžička, Jan Ježek, Andrea Hároniková, Martin Šiler, Pavel Zemánek, Veronika Holá

**Affiliations:** 1 Department of Microbiology, Faculty of Medicine and St. Anne’s Faculty Hospital, Brno 65691, Czech Republic; E-Mails: k.mlynarikova@gmail.com (K.M.); veronika.hola@fnusa.cz (V.H.); 2 Institute of Scientific Instruments of the Academy of Sciences of the Czech Republic, v.v.i., Královopolská 147, Brno 61264, Czech Republic; E-Mails: osamek@isibrno.cz (O.S.); berns@isibrno.cz (S.B.); jezek@isibrno.cz (J.J.); siler@isibrno.cz (M.Š.); pavlik@isibrno.cz (P.Z.); 3 Centre for Material Research, Brno University of Technology, Purkyňova 118, Brno 61200, Czech Republic; E-Mail: haronikova@fch.vutbr.cz

**Keywords:** Raman spectroscopy, bacteria, yeasts, culture media

## Abstract

Raman spectroscopy has a broad range of applications across numerous scientific fields, including microbiology. Our work here monitors the influence of culture media on the Raman spectra of clinically important microorganisms (*Escherichia coli*, *Staphylococcus aureus*, *Staphylococcus epidermidis* and *Candida albicans*). Choosing an adequate medium may enhance the reproducibility of the method as well as simplifying the data processing and the evaluation. We tested four different media per organism depending on the nutritional requirements and clinical usage directly on a Petri dish. Some of the media have a significant influence on the microbial fingerprint (Roosvelt-Park Institute Medium, CHROMagar) and should not be used for the acquisition of Raman spectra. It was found that the most suitable medium for microbiological experiments regarding these organisms was Mueller-Hinton agar.

## 1. Introduction

Raman spectroscopy is a physical method with a broad range of applications—it is suitable for the identification of substances, their structure and composition. The method is based on a non-destructive measurement of inelastic scattering of light (Raman scattering) that allows identification of a broad spectrum of molecules or characterization of molecular composition of a biological/non-biological sample by providing its spectroscopic fingerprint [1,2]. In last few years has been used for identification and description of biological systems ranging from biomolecules to whole cells and organisms [3,4,5,6,7,8,9,10,11,12,13,14,15,16,17,18,19,20].

The most frequent use of the Raman spectroscopy in microbiology is a phenotype measurement for strain identification (including bacteria, yeasts and algae) [2,3,11,13], but the method can be used to determine a presence of virulence factors in a certain strain, too [10,16,21]. Microorganisms can be analyzed directly from colonies grown on an agar plate [10,16], microcolonies on solid culture media [22] or single-cells obtained after a cultivation in liquid media [23]. As the Raman spectroscopy measures the molecular composition of the cells, spectra might be influenced by culture conditions.

Culture media differ in their composition to satisfy the nutritive requirements of a particular group of organisms as well as to serve as a selective and/or diagnostic tool. The aim of our study is to find suitable culture media (from commonly used media in the laboratories of clinical microbiology) for selected organisms by assessing limitations caused by the influence of culture media on the Raman fingerprint of the given strain. Thus reproducible spectra of microorganisms can be obtained which, in turn, simplifies the data processing and evaluation. The investigation presented here shows the influence of commonly used culture media on the Raman fingerprint of selected microorganisms belonging to the groups of Gram-positive bacteria, Gram-negative bacteria and yeasts—namely, *Staphylococcus aureus*, *Staphylococcus epidermidis*, *Escherichia coli*, and *Candida albicans.*

Staphylococci are Gram-positive (with a compact cell wall containing a thick peptidoglycan layer) cocci that tend to form irregular clusters. Most of the species belonging to the genus *Staphylococcus* colonize the skin plus mucous membranes of humans [24,25] and under certain conditions can cause infections [26]. The most clinically relevant species of the genus is *Staphylococcus aureus,* which can cause infectious diseases like impetigo, osteitis, cellulitis, boils, scalded skin syndrome, and septicemia, as well as a toxin-mediated food poisoning and a toxic shock syndrome [27]. When *S. aureus* enters the bloodstream, life-threatening invasive diseases, such as sepsis, osteomyelitis, endocarditis and meningitis can arise. These infections often have a nosocomial character [28] and are difficult to treat [28,29].

*Staphylococcus epidermidis*, the second most frequently isolated representative of staphylococci, is an important saprophytic organism found on the human skin. However, it can significantly contribute to ever-increasing morbidity and mortality of hospital-acquired infections [30]. Major infections caused by *S. epidermidis* are biofilm-associated medical-device-related infections. These include prosthetic valve endocarditis, urinary tract infections, central nervous system infections, eye infections as well as intravascular-catheter associated infections and septic loosening of joint prostheses after total joint arthroplasty [25,31,32,33,34,35,36,37].

*Escherichia coli* is a Gram-negative (having a more complicated cell-wall structure than the Gram-positive bacteria: the peptidoglycan layer is thin and sandwiched between the cytoplasmic membrane and bacterial outer membrane, and it contains phospholipids, lipopolysaccharides and proteins) rod that occurs in the intestinal tract of mammals as a common part of normal microflora. However, certain strains may cause infection in the human intestinal tract (e.g., toxin-producing strains). Moreover, *E. coli* is the most frequently isolated pathogen from community-acquired urinary tract infections as well as the most frequently isolated Gram-negative species from bloodstream infections [38].

*Candida albicans* still remains the most frequently isolated yeast from clinical specimens [39,40]. It is a polymorphic fungus that can switch phenotype between the unicellular yeast form and the filamentous form (hypha, pseudohypha) [41,42] that usually inhabits the mucosal membranes in humans. However, when the balance between *C. albicans* and host is disrupted, this yeast can cause various infections ranging from oral/superficial candidiasis and infections of the reproductive system to serious infections—invasive candidiasis and candidaemia, which are often life-threatening [43].

In the present study, we exploit point-by-point recording for significant parts of the microbial colonies grown on four different culture media (directly on the Petri dish) for each strain (selected media are commonly used in a microbiological laboratory for cultivation of the given organism). Specifically, we restricted the spot measurements to the central, middle and upper periphery of the microbial colony surfaces. We engaged the appropriate refocusing on a sample for each Raman spectra for the purpose of staying within the focal depth of the laser excitation and imaging optics.

## 2. Results and Discussion

As was mentioned above, in order to assess the influence of certain media on Raman spectra of microorganisms concerning the repeatability as well as the quality of specific fingerprints, we analyzed four organisms (*Staphylococcus aureus*, *Staphylococcus epidermidis*, *Escherichia coli* and *Candida albicans*), each on four culture media, as described further in Materials and Methods ion.

All culture media contain biomolecules so they can provide an adequate nutrient supply for the microorganisms to grow and reproduce. As microbial colonies are relatively small, a signal acquired from a microbial colony can also include a signal from culture medium when measuring the edges plus the molecules from the medium can be found in the volume of a colony (see Experimental Setup).

**Figure 1:**
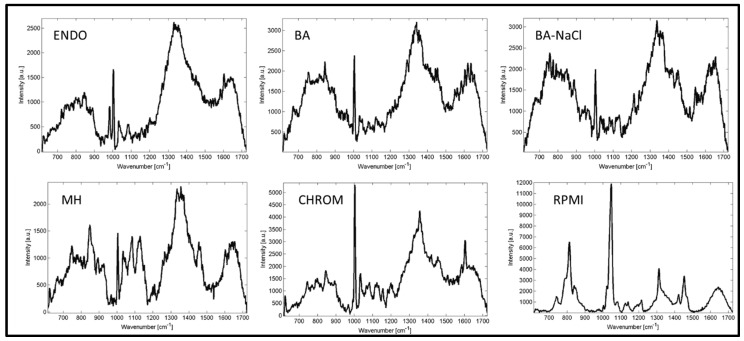
Raman spectra obtained from different culture media (ENDO—Endo agar, BA—blood agar; BA-NaCl—blood agar with 10% NaCl; MH—Mueller-Hinton agar; CHROM—CHROMagar Candida; RPMI—Roosvelt-Park Institute Medium 1640).

The Raman spectra of selected culture media are shown in Figure 1. Majority of tested media (Endo agar—ENDO, blood agar—BA, blood agar with 10% NaCl—BA-NaCl, Mueller-Hinton agar—MH) show weak intensity of peaks (ranging up to 2500–3000 a.u.). The other two media (CHROMagar Candida—CHROM and Roosvelt-Park Institute Medium 1640—RPMI) show strong signals with numerous detectable peaks with intensity up to 5000 a.u. for CHROM and 12,000 a.u. for RPMI. The intensity of the peaks detectable in microbial spectra ranges from approximately 2000 a.u. up to 20,000 a.u.

**Figure 2:**
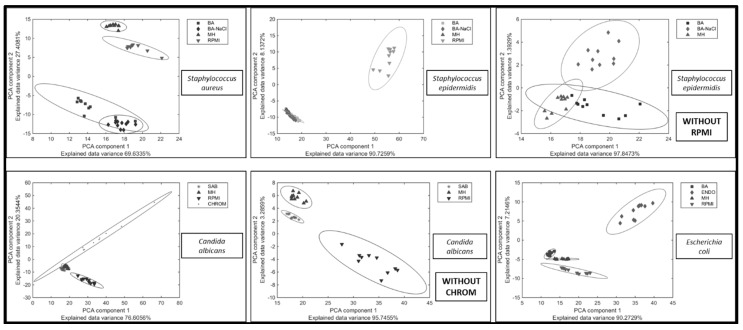
Repeatability of Raman spectra of the given organism after growth on different culture media (ENDO—Endo agar, BA—blood agar; BA-NaCl—blood agar with 10% NaCl; MH—Mueller-Hinton agar; CHROM—CHROMagar Candida; RPMI—Roosvelt-Park Institute Medium 1640). Plots marked “Without RPMI” and “Without CHROM” are displayed to simplify the comparison of variability of Raman spectra measurement on all of the media. The lower the variability within the species is, the smaller the area covered by an ellipsoid.

As we showed in our previous work, the overall repeatability of the specific Raman spectra associated with a certain microbial strain after repeated cultivation is very high, even for samples prepared more than a year apart [16]. To assess the repeatability for each organism-medium combination within the analysis we ran a principal component analysis (PCA) for all measurements of one organism grown on all corresponding media (Figure 2). All organisms were measured after identical cultivation conditions using the same equipment and exposition time.

Clustering of *S. aureus* spectra indicates that the most suitable medium for Raman spectral acquisition for this organism is MH medium. BA and BA-NaCl can also be used, but the repeatability of the fingerprint is lower. BA-NaCl is a selective medium, which could easily induce a dissociation of the strain (intra-strain variations) and therefore it might cause the variability in Raman spectra. A chemically well-defined medium containing many supplements, RPMI, induces a high variability in Raman spectra of *S. aureus* and therefore should not be used for analyses of this organism. Result for *Staphylococcus epidermidis* show the very same pattern.

Repeatability of *C. albicans* Raman spectra is high when using MH as well as SAB medium. On the other hand, cultivation on CHROM agar and RPMI medium induces a high variability of spectra. This might by influenced by different colony morphology induced by nutrients in a medium. For example, RPMI medium induces the hyphal form development leading to domination of this form over the yeast form [44].

**Figure 3:**
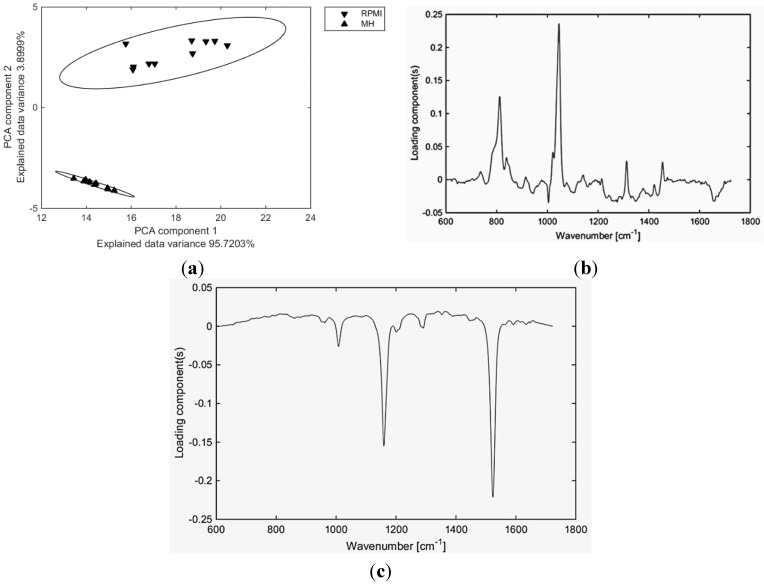
(**a**) Scores plot of the first two principal components relation for *E. coli* cultured on two different media—top cluster RPMI (down-triangle), bottom cluster MH (up-triangle). Using two principle components, one can clearly separate the clusters of spectra related to cultivation of the same microorganism on two different media. This suggests that during the growth of bacteria cultivated on RPMI agar, some substances from agar were absorbed into the volume of the colony translating to the final bacterial spectra; (**b**) Plot of loading of PC2 corresponding to Figure 3a supporting the contribution of spectral bands related to RPMI medium. Because of focusing conditions this is not due to the contribution from the underlying agar; (**c**) Plot of loading of PC2 corresponding to four bacteria included in our study cultured on the Mueller-Hinton agar supporting the contribution of spectral bands related to given microorganisms (three bands of beta-carotene). This is in contrast to Figure 3b where spectral bands related to RPMI medium were observed and shows that the Mueller-Hinton agar should be the medium of choice for microbiological experiments employing Raman spectroscopy measurements.

There is a low variability of Raman spectra obtained from *E. coli* colonies grown on MH agar, too. Cultivation on BA agar is shown to be comparable to MH, concerning the variability of spectra. *E. coli* fingerprint repeatability on ENDO agar is, however, lower than on previously mentioned media. This might be caused by the presence of a dye (fuchsine) in the medium. Fuchsine is relatively toxic for bacteria and can induce a dissociation of the given strain. Equally as in staphylococci, RPMI medium induces the high variability of Raman spectra in *E. coli* and therefore the repeatability of measurement on this medium is not sufficient. Figure 3 shows the comparison of variability in *E. coli* spectra from RPMI and MH as an example of best-case scenario and worst-case scenario cultivation media for Raman spectral acquisition from bacterial colonies. For comparison, Figure 3c includes a plot of loading for the best-case scenario for all tested microorganisms—cultivation on MH agar.

**Figure 4:**
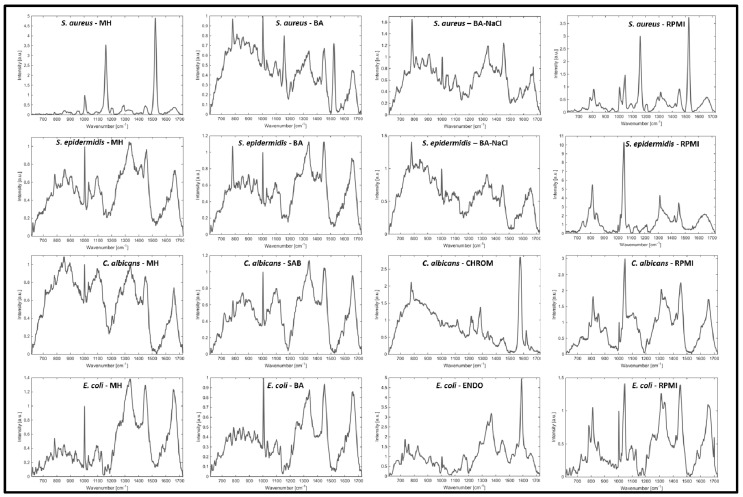
Normalized representative Raman spectra of the microorganisms on all used culture media (ENDO—Endo agar, BA—blood agar; BA-NaCl—blood agar with 10% NaCl; MH—Mueller-Hinton agar; CHROM—CHROMagar Candida; RPMI—Roosvelt-Park Institute Medium 1640).

More detailed analysis of Raman spectra corresponding to the selected organisms show that the fingerprint of RPMI medium (here RPMI appeared as a worst-case scenario medium) is visible in the spectrum of microbial colonies (detectable peaks with high intensity at approx. 820 cm^−1^, 1050 cm^−1^, 1310 cm^−1^ and 1450 cm^−1^) (Figure 4). Consequently, such a high influence of RPMI medium on the Raman spectra of selected microorganisms translates to the large size of the area given by the Mahalanobis distance (Figure 2 and Figure 3). Also high influence of these peaks can be a trace in dedicated PC-loadings (each PCA can be further analyzed using the PC-loadings) when using PCA analysis for the selected samples (Figure 3). This is in contrary to MH agar (see Figure 3c) which contribution to PCA analysis of given microorganisms is very small—it is also translated to small area of ellipsoids calculated using Mahalanobis distance.

This problem (as with RPMI medium) is also observed when using CHROMagar (detectable peaks at approx. 1040 cm^−1^, 1360 cm^−1^ and 1600 cm^−1^).

Thus, detection of medium-induced peaks in Raman spectra of microorganisms impairs the association of the spectra with the certain corresponding organism as well as the specificity of microbial fingerprint and therefore should be avoided.

In addition, the spectra of each organism slightly change with the used culture medium. This might be a result of different nutrients intake leading to the differences in cell and colony composition.

According to the results presented above, the most suitable medium for analyses of all tested bacterial strains is MH agar. Moreover, analyses of colonies grown of MH agar show a very good repeatability also for the yeast strains. This within-analysis repeatability of MH agar used for yeast strains are consistent with our recent work employing *Candida parapsilosis* strains [16]. In addition to MH agar, SAB agar is shown to provide the comparable repeatability of the yeast strain measurement.

## 3. Materials and Methods

### 3.1. Microorganisms and Sample Preparation

Selected organisms representing Gram-positive bacteria (*Staphylococcus aureus* D 47, *Staphylococcus epidermidis*, STO 60), Gram-negative bacteria (*Escherichia coli*, CCM 3988) and yeasts (*Candida albicans*, CCM 8261) were used in the study. The CCM strains were obtained from the Czech Collection of Microorganisms. Both staphylococcal strains were isolated from clinical material and stored in the Culture Collection of the Department of Microbiology, St. Anne’s Faculty Hospital in Brno, Czech Republic. All of these strains were stored at –70 °C. Before the experiment, the strains were thawed quickly at the room temperature and cultivated on the Mueller-Hinton agar (MH, Oxoid, Basingstoke, UK) at 37 °C for 24 h. Grown colonies were consequently transferred onto certain solid media in Petri dishes: MH, Blood agar with sheep erythrocytes (BA), Blood agar with sheep erythrocytes and 10% NaCl (BA-NaCl), Sabouraud agar (SAB, Merck, Germany), Roosvelt-Park Institute Medium 1640 with L-glutamine (RPMI, Sigma Aldrich, Munich, Germany) and CHROMagar Candida (CHROM, CHROMagar Company, Paris, France), Endo agar (ENDO, Oxoid, Basingstoke, UK), with each organism cultured on four media (see Table 1). Selected media are commonly used in clinical laboratories. However, selected organisms have different nutritive demands and therefore not all of the media are suitable for all of the organisms and can inhibit their growth (Table 1).

The cultivation on selected media before the experiment ran for 24 h (except for *Candida albicans* on CHROMagar, 48 h) at 37 °C.

**Table 1:** Cultivation of microorganisms using different culture media (x = used for the given organism).

Medium/Organism	*Staphylococcus aureus*	*Staphylococcus epidermidis*	*Escherichia coli*	*Candida albicans*
Mueller-Hinton agar	x	x	x	x
Blood agar	x	x	x	
Blood agar-NaCl	x	x		
CHROMagar Candida				x
Roosvelt-Park Institute Medium 1640 with L-glutamine	x	x	x	x
Sabouraud agar				x
Endo agar			x	

### 3.2. Experimental Setup

**Figure 5:**
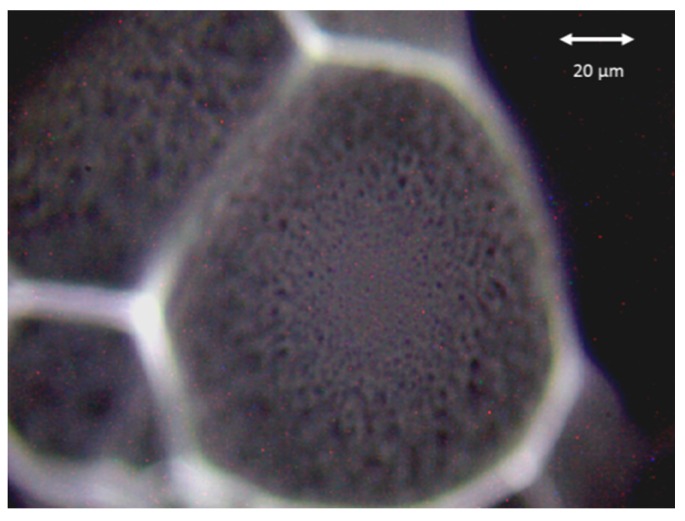
Image of a colony of *Staphylococcus epidermidis*.

Grown microbial colonies were examined using a commercial Renishaw Raman spectrometer (Renishaw inVia Raman Spectrometer, Renishaw plc., Wotton-under-Edge, UK), with 785 nm single-mode diode laser as the excitation source. After cultivation of 24 h on MH agar the colony size was approximately 100 µm so that spectra could be taken easily with focused laser (diameter of laser is approx. 2 μm × 10 μm). The steep decrease in colony height outside the flat center of a growing colony (here the diameter is approx. 40 μm). This is visualized by unfocused colony surface at the periphery of the colony (Figure 5). Note that the displayed colony is relatively small and was chosen just to demonstrate the shape of a bacterial colony. Mature colonies can be as large as a few mm in diameter with estimated thicknesses of hundreds of micrometers. We estimated the height of the displayed colony at the distance of 15 μm from the bottom of the colony by refocusing laser spot on the sample at 30 μm and the flat center on the top of the colony at 110 μm.

A laser beam was focused onto a sample by the microscope objective (Leica, Wetzlar, Germany, 50×, NA (Numerical aperture) 0.5) with the laser spot diameter of approximately 2 μm × 10 μm (note that such laser spot shape is characteristic for the Renishaw inVia instrument), with full axial depth of the excitation region at 8 µm [45]. As the laser was focused onto a surface of the particular colony, we measured the response of a small fraction of a colony directly grown on the culture medium in the petri dish as recently described in the work of Samek *et al.* [16]. To reliably apply the Raman technique, the laser was not focused at the area near the periphery where the height of the colony falls steeply. In this way, the signal contribution from the underlying agar can be minimized—the signal which originates from the culture medium is very low and not influencing the Raman spectra from a colony.

Overview spectra were acquired in the range of 600–1700 cm^−1^. As the interfering signal from the culture medium may vary depending on actual colony proportions, each spectrum was measured for 15 s from different parts of a colony in a total of 10 measurements for one strain (spectra were obtained from at least 3 colonies per strain).

### 3.3. Data Analysis

The Raman spectra were treated with the Savitzky-Golay coupled advanced rolling filter background removal routine (see [13]), and subsequently analyzed using the standard multivariate principle component program written in-house using MatLab software (MathWorks, Natick, MA, USA). Spectra shown at Figure 2 were normalized to the peak assigned to the amino acid phenylalanine [12] at approximately 1004 cm^−1^. Phenylalanine is present in all of the tested microorganisms and was used for standardization following numerous tests to use this peak intensity. This is not ideal, however, this procedure could be used in instances where no proper standards available [12]. The groups were marked by ellipsoids with Mahalanobis distance of 3 [46].

## 4. Conclusions

We analyzed four microorganisms (*Staphylococcus aureus*, *Staphylococcus epidermidis*, *Escherichia coli* and *Candida albicans*), each grown on four different culture media, by Raman spectroscopy to find which medium influences the Raman spectrum fingerprint of each organism less. We showed that Mueller-Hinton agar is the most suitable medium for analyses of all tested microorganisms (compared with blood agar, blood agar with 10% NaCl, Roosvelt-Park Institute Medium 1640, Endo agar and CHROMagar Candida) providing a good repeatability of the microbial fingerprint as well as a low variability of spectra within the analysis. Contrariwise, the Roosvelt-Park Institute Medium 1640 and the CHROMagar Candida were shown to impair the specific microbial fingerprint by introducing the medium-specific peaks into it and subsequently resulting in variable within-analysis spectra and low repeatability of the measurement.

## References

[1] Schie I.W., Huser T. (2013). Methods and applications of Raman microspectroscopy to single-cell analysis. Appl. Spectrosc..

[2] Read D.S., Whiteley A.S. (2015). Chemical fixation methods for Raman spectroscopy-based analysis of bacteria. J. Microbiol. Methods.

[3] Maquelin K., Kirschner C., Choo-Smith L.P., Ngo-Thi N.A., van Vreeswijk T., Stammler M., Endtz H.P., Bruining H.A., Naumann D., Puppels G.J. (2003). Prospective study of the performance of vibrational spectroscopies for rapid identification of bacterial and fungal pathogens recovered from blood cultures. J. Clin. Microbiol..

[4] Afseth N.K., Bloomfield M., Wold J.P., Matousek P.A. (2014). Novel approach for subsurface through-skin analysis of salmon using spatially offset raman spectroscopy (SORS). Appl. Spectrosc..

[5] Notingher I. (2007). Raman spectroscopy cell-based biosensors. Sensors.

[6] Almarashi J.F.M., Kapel N., Wilkinson T.S., Telle H.H. (2012). Raman spectroscopy of bacterial species and strains cultivated under reproducible conditions. Spectrosc. Int. J..

[7] De Gelder J., de Gussem K., Vandenabeele P., Moens L. (2007). Reference database of Raman spectra of biological molecules. J. Raman Spectrosc..

[8] Martinelli A. (2014). Effects of a protic ionic liquid on the reaction pathway during non-aqueous sol-gel synthesis of silica: A Raman spectroscopic investigation. Int. J. Mol. Sci..

[9] Brauchle E., Schenke-Leyland K. (2013). Raman spectroscopy in biomedicine—Non-invasive *in vitro* analysis of cells and extracellular matrix components in tissues. Biotechnol. J..

[10] Samek O., Al-Marashi J.F.M., Telle H.H. (2010). The potential of Raman spectroscopy for the identification of biofilm formation by *Staphylococcus epidermidis*. Laser Phys. Lett..

[11] Samek O., Telle H.H., Harris L.G., Bloomfield M., Mack D. (2008). Raman spectroscopy for rapid discrimination of *Staphylococcus epidermidis* clones related to medical device-associated infections. Laser Phys. Lett..

[12] Bernatová S., Samek O., Pilát Z., Šerý M., Ježek J., Jákl P., Šiler M., Krzyžánek V., Zemánek P., Holá V. (2013). Following the mechanisms of bacteriostatic *versus* bactericidal action using Raman spectroscopy. Molecules.

[13] Samek O., Jonáš A., Pilát Z., Zemánek P., Nedbal L., Tříska J., Kotas P., Trtílek M. (2010). Raman microspectroscopy of individual algal cells: Sensing unsaturation of storage lipids *in vivo*. Sensors.

[14] Sandt C., Smith-Palmer T., Pink J., Brennan L., Pink D. (2007). Confocal Raman microspectroscopy as a tool for studying the chemical heterogeneities of biofilms *in situ*. J. Appl. Microbiol..

[15] Choo-Smith L.P., Marquelin K., van Vreeswijk T., Bruining H.A., Puppels G.J., Ngo Thi N.A., Kirchner C., Naumann D., Ami D., Villa A.M. (2001). Investigating microbial (Micro)colony heterogeneity by vibrational spectroscopy. Appl. Environ. Microbiol..

[16] Samek O., Mlynariková K., Bernatová S., Ježek J., Krzyžánek V., Šiler M., Zemánek P., Růžička F., Holá V., Mahelová M. (2014). *Candida parapsilosis* Biofilm Identification by Raman Spectroscopy. Int. J. Mol. Sci..

[17] Maquelin K., Choo-Smith L.P., van Vreeswijk T., Endtz H.P., Smith B., Bennett R., Bruining H.A., Puppels G.J. (2000). Raman Spectroscopic Method for Identification of Clinically Relevant Microorganisms Growing on Solid Culture Medium. Anal. Chem..

[18] Maquelin K., Choo-Smith L.P., Endtz H.P., Bruining H.A., Puppels G.J. (2002). Rapid identification of *Candida* species by confocal Raman microspectroscopy. J. Clin. Microbiol..

[19] Almarashi J.F.M., Kapel N., Wilkinson T.S., Telle H.H. (2013). Raman spectroscopy of bacterial species and strains cultivated under reproducible conditions. Advances in Biomedical Spectroscopy.

[20] Espagnon I., Ostrovskii D., Mathey R., Dupoy M., Joly P.L., Novelli-Rousseau A., Pinston F., Gal O., Mallard F., Leroux D.F. (2014). Direct identification of clinically relevant bacterial and yeast microcolonies and macrocolonies on solid culture media by Raman spectroscopy. J. Biomed. Opt..

[21] Wulf M.W.H., Willemse-Erix D., Verduin C.M., Puppels G., van Belkum A., Maquelin K. (2012). The use of Raman spectroscopy in the epidemiology of methicillin-resistant *Staphylococcus aureus* of human- and animal-related clonal lineages. Clin. Microbiol. Infect..

[22] Mathey R., Dupoy M., Espagnon I., Leroux D., Mallard F., Novelli-Rousseau A. (2015). Viability of 3 h grown bacterial micro-colonies after direct Raman identification. J. Microbiol. Methods.

[23] Schuster K.C., Urlaub E., Gapes J.R. (2000). Single-cell analysis of bacteria by Raman microscopy: Spectral information on the chemical composition of cells and on the heterogeneity in a culture. J. Microbiol. Methods.

[24] Vandenbergh M.F., Verbrugh H.A. (1999). Carriage of *Staphylococcus aureus*: Epidemiology and clinical relevance. J. Lab. Clin. Med..

[25] Piette A., Verschraegen G. (2009). Role of coagulase-negative staphylococci in human disease. Vet. Microbiol..

[26] Kocianova S., Vuong C., Yao Y., Voyich J.M., Fischer E.R., DeLeo F.R., Otto M. (2005). Key role of poly-g-DL-glutamic acid in immune evasion and virulence of *Staphylococcus epidermidis*. J. Clin. Investig..

[27] Lindberg E., Adlerberth I., Matricardi P., Bonanno C., Tripodi S., Panetta V., Hesselmar B., Saalman R., Åberg N., Wold A.E. (2011). Effect of lifestyle factors on *Staphylococcus aureus* gut colonization in Swedish and Italian infants. Clin. Microbiol. Infect..

[28] Van den Berg S., Bonarius H.P.J., van Kessel K.P.M., Elsinga G.S., Kooi N., Westra H., Bosma T., van der Kooi-Pol M.M., Koedijk D.G.A.M., Groen H. (2015). A human monoclonal antibody targeting the conserved staphylococcal antigen IsaA protects mice against *Staphylococcus aureus* bacteremia. Int. J. Med. Microbiol..

[29] Cosgrove S.E. (2006). The relationship between antimicrobial resistance and patient outcomes: Mortality, length of hospital stay, and health care costs. Clin. Infect. Dis..

[30] McCann M.T., Gilmore B.F., Gorman S.P. (2008). *Staphylococcus epidermidis* device-related infections: Pathogenesis and clinical management. J. Pharm. Pharmacol..

[31] Verhoef J., Fleer A. (1983). *Staphylococcus epidermidis* endocarditis and *Staphylococcus epidermidis* infection in an intensive care unit. Scand. J. Infect. Dis. Suppl..

[32] Jansen B., Hartmann C., Schaumacher-Pedreau F., Peters G. (1991). Late onset endopthalmitis associated with intraocular lens: A case of molecularly proved *S. epidermidis* aetiology. Br. J. Ophthalmol..

[33] Warren J.W. (2001). Catheter-associated urinary tract infection. Int. J. Antimicrob. Agents.

[34] Rupp M.E., Archer G.L. (1994). Coagulase-negative staphylococci: Pathogens associated with medical progress. Clin. Infect. Dis..

[35] Rupp M.E., Hamer K.E. (1998). Effect of subinhibitory concentrations of vancomycin, cefazolin, ofloxacin, L-ofloxacin and D-ofloxacin on adherence to intravascular catheters and biofilm formation by Staphylococcus epidermidis. J. Antimicrob. Chemother..

[36] Gallo J., Kolar M., Novotny R., Rihakova P., Ticha V.V. (2003). Pathogenesis of prosthesis-related infection. Biomed. Pap. Med. Fac. Univ. Palacky Olomouc Czech. Repub..

[37] Ip D., Yam S.K., Chen C.K. (2005). Implications of the changing pattern of bacterial infections following total joint replacements. J. Orthop. Surg..

[38] Riley L.W. (2014). Pandemic lineages of extraintestinal pathogenic *Escherichia coli*. Clin. Microbiol. Infect..

[39] Trofa D., Gácser A., Nosanchuk J.D. (2008). *Candida parapsilosis*, an emerging fungal pathogen. Clin. Microbiol. Rev..

[40] Hattori H., Iwataa T., Nakagawa Y., Kawamoto F., Tomitaa Y., Kikuchi A., Kanbe T. (2006). Genotype analysis of *Candida albicans* isolates obtained from different body locations of patientswith superficial candidiasis using PCRs targeting 25S rDNA and ALT repeat sequences of the RPS. J. Dermatol. Sci..

[41] Lim C.S.Y., Rosli R., Seow H.F., Chong P.P. (2012). *Candida* and invasive candidiasis: Back to basics. Eur. J. Clin. Microbiol. Infect. Dis..

[42] Machová E., Fiačanová L., Čížová A., Korcová J. (2015). Mannoproteins from yeast and hyphal form of *Candida albicans* considerably differ in mannan and protein content. Carbohydr. Res..

[43] Yan L., Yang C., Tang J. (2013). Disruption of the intestinal mucosal barrier in *Candida albicans* infections. Microbiol. Res..

[44] Chandra J., Kuhn D.M., Mukherjee P.K., Hoyer L.L., McCormick T., Ghannoum M.A. (2001). Biofilm formation by the fungal pathogen *Candida albicans*: Development, architecture, and drug resistance. J. Bacteriol..

[45] (2003). Renishaws EasyConfocal Raman Method. Technology Note from the Spectroscopy Products Division.

[46] De Maesschalck R., Jouan-Rimbaud D., Massart D.L. (2000). The Mahalanobis distance. Chemometr. Intell. Lab..

